# Elaboration of an educational video for cardiopulmonary resuscitation with chest compressions in adults

**DOI:** 10.1590/0034-7167-2022-0367

**Published:** 2023-10-09

**Authors:** Rafael de Lima Carmo, Evandro Luiz Panho, Christian Antônio Zago de Quadros, João Vitor Antunes Lins dos Santos, Érica de Brito Pitilin, Jeferson Santos Araújo, Rosana Aparecida Spadoti Dantas, Vander Monteiro da Conceição

**Affiliations:** I Universidade Federal da Fronteira Sul. Chapecó, Santa Catarina, Brazil; II Universidade de São Paulo. Ribeirão Preto, São Paulo, Brazil

**Keywords:** Out-of-Hospital Cardiac Arrest, Cardiopulmonary Resuscitation, Instructional Film and Video, Validity Study, Public Health Nursing., Paro Cardíaco Extrahospitalario, Reanimación Cardiopulmonar, Película y Video Educativos, Estudio de Validación, Enfermería en Salud Pública., Parada Cardíaca Extra-Hospitalar, Reanimação Cardiopulmonar, Filme e Vídeo Educativo, Estudo de Validação, Enfermagem em Saúde Pública

## Abstract

**Objectives:**

to develop and analyze the face and content validity of a storyboard for constructing an educational video for training laypersons in cardiopulmonary resuscitation with only chest compressions in adults.

**Methods:**

a methodological study of storyboard elaboration and validity for producing an educational health video. The storyboard was submitted to analysis of 20 judges to assess its adequacy with the proposed objective. To assess the agreement between judges, the Content Validity Index was calculated. After validating the storyboard, video production took place.

**Results:**

the Content Validity Index met expectations. Its mean in each group was ≥ 0.90 (lay judges = 0.97; expert judges = 0.90; all judges = 0.94).

**Conclusions:**

the video produced is composed of scientific information, professional expertise and laypersons’ perceptions, making it important evidence for health education.

## RESUMO

**Objetivos:**

elaborar e analisar a validade de face e de conteúdo de um *storyboard* para a construção de um vídeo educativo para treinamento de leigos em reanimação cardiopulmonar somente com compressões torácicas em adultos.

**Métodos:**

estudo metodológico de elaboração e validação de um *storyboard* para a produção de um vídeo educativo em saúde. O *storyboard* foi submetido à análise de 20 juízes, para avaliação de sua adequação com objetivo proposto. Para avaliar a concordância entre os juízes, calculou-se o Índice de Validade de Conteúdo. Após a validação do *storyboard*, ocorreu a produção do vídeo.

**Resultados:**

o Índice de Validade de Conteúdo obtido atendeu ao esperado. A média dele em cada grupo esteve ≥ 0,90 (juízes leigos = 0,97; juízes experts = 0,90; todos os juízes = 0,94).

**Conclusões:**

o vídeo produzido é composto por informações científicas, expertise profissional e percepções de leigos, tornando-se importante evidência para a educação em saúde.

## RESUMEN

**Objetivos:**

elaborar y analizar la validez de apariencia y contenido de un storyboard para la construcción de un video educativo para la formación de legos en reanimación cardiopulmonar con solo compresiones torácicas en adultos.

**Métodos:**

estudio metodológico de la elaboración y validación de un storyboard para la producción de un video educativo en salud. El storyboard fue sometido al análisis de 20 jueces, para evaluar su adecuación al objetivo propuesto. Para evaluar la concordancia entre los jueces se calculó el Índice de Validez de Contenido. Después de validar el guión gráfico, se produjo el video.

**Resultados:**

el Índice de Validez de Contenido obtenido cumplió con las expectativas. Su promedio en cada grupo fue ≥ 0,90 (jueces legos = 0,97; jueces expertos = 0,90; todos los jueces = 0,94).

**Conclusiones:**

el video producido está compuesto por información científica, experiencia profesional y percepciones de los legos, lo que lo convierte en evidencia importante para la educación en salud.

## INTRODUCTION

Cardiorespiratory arrest is a public health problem that affects people all over the world, characterized by the sudden interruption of cardiac and respiratory function and lack of awareness^(1)^. Although data on cardiopulmonary arrest in Brazilian health services are scarce, there is information that approximately 200,000 cases occur per year and that more than half of them occur outside the hospital^(2)^.

In the out-of-hospital environment, the American Heart Association (AHA) emphasizes the importance of care by trained laypeople in the identification and immediate initiation of cardiopulmonary resuscitation in cases of cardiac arrest. The percentage of adults undergoing cardiopulmonary resuscitation is less than 40% and needs to be increased. It is recommended that, even if the signs of cardiac arrest are unclear, a layperson should initiate cardiopulmonary resuscitation with chest compressions only. People who are not health professionals do not experience cases of cardiorespiratory arrest on a daily basis during their lives and, therefore, have difficulty identifying the victim’s arterial pulse. Therefore, the AHA indicates cardiopulmonary resuscitation with chest compressions only, as there is evidence that damage is less when an individual receives chest compressions without being in cardiorespiratory arrest than one who does not receive chest compressions and is^(3)^. Thus, members of civil society should receive training in cardiopulmonary resuscitation in order to enable them to recognize and manage cardiorespiratory arrest, increasing the chances of survival of out-of-hospital victims.

Educational practice is a present and constant element in everyday social life, as routinely people teach and learn something. In the meantime, nurses are important mediating agents for health information acquisition, which is easy to understand and based on science. By providing information, nurses enable recipients to acquire new skills, empowerment and acting with social responsibility^(4)^, such as in caring for an individual in cardiac arrest.

Currently, considering the technological advances incorporated in society, nurses must use innovative learning tools that are rapidly disseminated and capable of promoting health in different scenarios, such as training lay people, without neglecting the scientific content of the information offered^(5)^. Educational video production and information dissemination using this audiovisual resource arouses curiosity in the public, in addition to making learning more sophisticated in relation to the themes addressed^(6)^. Therefore, educational videos have been used in different contexts and have shown good results in health education.

## OBJECTIVES

To develop and analyze the face and content validity of a storyboard for constructing an educational video for training laypersons in cardiopulmonary resuscitation with only chest compressions in adults.

## METHODS

### Ethical aspects

The study was approved by the Research Ethics Committee, respecting the ethical precepts of Resolution 466/12 of the Brazilian National Health Council. In the e-mail, with an invitation and the Informed Consent Form (ICF), candidates were duly informed about their full freedom to decide about their participation as well as their freedom to give up at any time and withdraw their consent, guaranteed the exemption from any form of penalty. All participants voluntarily agreed by checking the alternative “Yes, I accept” of the digital ICF.

### Study design, period and place

This is a methodological study of creation and validity of storyboard face and content for producing a video for use in health education. This tool was developed according to the following production phases of an educational video: pre-production, production and post-production^(7)^. Due to its nature, the study was not based on the guidelines inserted in the Enhancing the Quality and Transparency of health Research (EQUATOR) network for the main investigations in the health area.

The study was carried out from April 2021 to May 2022 virtually (online), in the pre-production phase, and in person (study headquarters university), in the production and post-production phases.

### Study protocol

#### Pre-production phase: storyboard elaboration and validity by judges

A storyboard based on the AHA guidelines^(8)^ was prepared, considering that the referred institution is a reference for training lay people and health professionals in cardiopulmonary resuscitation. The storyboard created features 14 scenes divided into three introductory scenes, 10 instructional scenes and an ending scene. This construction stage took place between April and June 2021.

After preparing the storyboard, it was sent for face and content validity by judges. In this investigation, it is considered that content validity signals to researchers the degree of adjustment between the knowledge presented and the thematic area of interest, and face validity indicates whether the prepared material is understandable by the reader or people from the target population^(9)^. Therefore, for the proposed validity, two groups of judges were selected: laypersons (target population of interest) and experts (professional specialists in the subject). According to the framework adopted, 10 lay judges and 10 expert judges participated in the research, surpassing the recommendation of a minimum number of six judges for the aforementioned validity^(9)^. This framework has been adopted by other researchers in validity studies of educational materials^(10-11)^. Assessment took place from October 2021 to March 2022.

For storyboard face and content validity, the group of lay judges was formed by people from the social context of the researchers, using the snowball technique, and who met the following inclusion criteria: adults, regardless of sex and who reported no have prior knowledge about cardiopulmonary resuscitation.

The group of expert judges was composed of individuals who met the following inclusion criteria: health professionals (doctors or nurses); specialists in emergency and/or intensive care and/or cardiology, proven by certification provided by Higher Education Institution. Specialization could be replaced if the judge had at least five years of professional experience in critical patient care.

These individuals received a standardized invitation, sent by email, and when they agreed to participate in the study they were directed, via link, to a form on Google Forms^®^, in which they expressed their consent to the ICF.

Subsequently, each judge answered a sociodemographic form (variables for lay judges: age, sex, level of education and employment relationship; variables for expert judges: age, sex, degree, year of graduation, length of professional experience, specialty and field of acting). All 20 individuals approached agreed to participate.

Lay judges aimed at assessing the language used and the systematization of the information offered, while expert judges had the intention of judging, in addition to the characteristics assessed by laypersons, the cardiopulmonary resuscitation instructional content. Each judge assessed the 14 scenes using a form. For each scene, there are two evaluative steps, and in the first they responded to the command “assess the scene for adequacy and clarity of its content”, with a single Likert-type answer: “not clear “, “little clear”, “fairly clear” and “very clear”. For the assessment process, we did not use the terms “face validity” and “content validity”, because we considered that the 10 expert judges were chosen for their knowledge in the area addressed in the storyboard and not for methodological studies of tool validity, such as measurement scales or educational materials, which also applied to lay judges. In the second stage, participants could submit suggestions for textual improvement and/or correction by filling in the field “leave your suggestion about the scene”.

Judges’ suggestions were discussed in a research team meeting, in March 2022, and accepted when consistent with the study proposal. Suggestions that were not accepted were justified with scientific literature. Thus, at this stage, the storyboard was validated, which was used in video production, a phase described below.

#### Production phase

For recording, seven people were needed, three actors, one cameraman and three extras. Among the three actors, only one is not part of the research team, and he agreed with the assignment of his image rights registered by his signature in a specific document.

On the day of recording, the entire team met and carried out the following steps: 1) Reading and re-reading of validated storyboard, in order to memorize the lines and scenes; Recognition of the space in which recording would take place and marking the positioning of actors and extras on the set; 3) Selection of the necessary materials for composing the scene, which were obtained in the laboratory of semiology and semiotics at the university; 4) Actors rehearsal; 5) First recording to confirm the cameraman’s position and obtain the best recording angle; 6) Official scene recording. The time spent for the described steps was four hours.

In the official recording stage, 17 videos were produced alluding to the 14 storyboard scenes. Images were recorded using a video recording application of an iPhone^®^ XR. At each recording, the scene was reviewed by the research team, and after approval, the scene was moved on to the next scene. Scene 5 “scene security” and scene 14 “ending” were shot twice due to environmental interference.

The material produced was added to a digital folder accessible only to the research team on Google Drive^®^. In this same virtual environment, 14 audios were archived, which were recorded for association with the images obtained (scene narration). They were produced by a research member, on the same mobile phone, now in an audio recording application. All material was archived until the post-production phase.

#### Post-production phase

This phase was developed by a nurse with technical training in informatics and a member of the research team in May 2022. The editing process was carried out using software from the Adobe^®^ package (license acquired by the research team). The previously recorded scenes were imported into Adobe After Effects^®^ and grouped into “compositions”. Each of them was numbered according to the storyboard sequence, and then associated with the narration audio, image and subtitle. The scenes that had a shorter duration than the narration audio were frozen in the last image chart, in order to contemplate all the information offered. At the end, there were 14 grouped compositions.

For image editing, the fifteenth composition was created (association of the previous 14) and a LUT was used, a technique used in image processing and treatment in order to maintain all scenes with standard lighting and shading. In audio processing, the final composition was imported into Adobe Audition^®^, performing file equalization and noise removal.

The final composition, with image and audio treated, was added to Adobe Media Encoder^®^ for rendering, being saved with the following output parameters: Mp4 H.264 with 1920x1080p resolution.

#### Analysis of results, and statistics

The researchers transferred judges’ responses to a database in Microsoft Excel^®^, validated by double typing. Subsequently, data were transferred and analyzed using the Statistical Package for the Social Sciences^®^ (SPSS), version 20.0. Data from judges’ sociodemographic variables were analyzed descriptively.

For storyboard face and content validity, the Content Validity Index was calculated considering judges’ answers in the assessment of each of the 14 scenes presented. According to the framework used^(12)^, answers “fairly clear” and “very clear” were considered for the calculation, and the values obtained should not be less than 0.78 in a survey with more than six judges. The results were presented in tables and described in text.

## RESULTS

In Chart 1, there are descriptions of the 14 built scenes, which were divided into three introductory scenes, 10 instructional scenes and a final scene, constituting the storyboard already submitted to face and content validity by judges.

**Chart 1:** Storyboard after validity, Chapecó, Santa Catarina, Brazil, 2022

Scene	Scene description	Scene content
1	Introduction 1	Narrated text (black background): “Did you know that for every minute of cardiac arrest a 7% chance of survival is lost?”. Then the text disappears and a quick video appears of an actor (member of the research team) simulating sudden illness and after an ambulance with siren and light bars turned on with difficulty moving in busy traffic.
2	Introduction 2	Narrated text (black background): “In some cases, even help arrives...”. Then the text disappears and there is a scene of the emergency team covering a corpse.
3	Introduction 3	Narrated text (black background): “You can change this fate!”. Narrated text: Educational Video for Training in Cardiopulmonary Resuscitation Using Only Chest Compressions in Adults (*Vídeo Educativo para Treinamento em Reanimação Cardiopulmonar Somente com Compressões Torácicas em Adultos*), according to the American Heart Association guidelines.
4	Emergency identification	The scene starts with two friends (Actor 1 and Actor 2) taking a walk in the open air. When crossing the security strip, Actor 1 suddenly falls. This moment there is a trained layperson (Actor 3) in chest compressions at the bus stop next door.
5	Scene security	Actor 3, leading the scene, addresses two pedestrians (extra) in a clear tone, and says “I need you to stop traffic so I can help”. Then, pedestrians wave to cars requesting the vehicle to stop. (Quick scene - while traffic is stopped, Actor 3 goes to the victim). (Appears in the video, in text/narration format: 1^st^ step: ensure the place safety so that a second accident does not occur).
6	Call for help 1	Immediately, Actor 3 turns to Actor 2 and says, “Hey you, call SAMU 192 (ambulance) and ask for help at this address, and request an AED”. (In text/narration format: Step 2: ask for help and start chest compressions)
7	Call for help 2	The camera focuses on the call, and the victim (Actor 1) and Actor 3 appear in the background. Actor 2 calls SAMU 192, “My name is Lucas, my friend fainted, he’s unconscious and not breathing, it’s here at UFFS, in front of the bus stop next to the laboratories”. (While the call is taking place, the following text/narration appears in the video: when calling emergency, keep calm, say your name, the number of people involved and what happened and the exact location with reference point)”.
8	Responsiveness + breathing movements	When approaching patients (Actor 1) over safety strip, Actor 3 calls out to him and says, “Sir, sir, can you hear me?”. At the same time, Actor 3 places his hands on Actor 1’s shoulders and performs a light shake, and there is no response. Then he proceeds to check for breathing movements. He also does not identify them. (Appears in text/narration format in the video: 3^rd^ step: identify if the victim has any reaction, if they breathe, no reaction + no breathing = start cardiopulmonary resuscitation). At this moment, Actor 3 realizes that Actor 1 is in a probable cardiorespiratory arrest.
9	Cardiopulmonary resuscitation 1	Actor 3 then kneeling next to the victim initiates cardiopulmonary resuscitation, demonstrating with pauses in each scene of the video and written/narrated guidance: Moment 1: positioning hands (Text/narration: overlap your hands and interlace your fingers and place the “heel” of your dominant hand over the center of the victim’s chest).
10	Cardiopulmonary resuscitation 2	Moment 2: Actor 3 positions himself to start cardiopulmonary resuscitation (Text/narration: project your torso over the victim leaving arms and elbows extended, without flexing them).
11	Cardiopulmonary resuscitation 3	Moment 3: Actor 3 starts compressions (Text/narration: using your weight, forcefully compress the chest, at least 5 cm deep, always returning compressions until the chest returns to normal position).
12	Cardiopulmonary resuscitation 4	Moment 4: Actor 3 continues with continuous compressions (Text/narration: compress 100 to 120 times per minute, without interrupting, until help arrives).
13	Cardiopulmonary resuscitation 5	Moment 5: change of rescuer (Actor 3) with Actor 2 after he has already asked for help (Text: when you get tired, take turns with someone else. Changing quickly, without taking more than 10 seconds).
14	Finalization	A specialized team arrives at the location and with blurring, only the title of the research becomes visible. (Text/narration: “Educational video for training in cardiopulmonary resuscitation with chest compressions only in adults”). After the title leaves the screen, the search credits appear.

For the final version of the storyboard, as presented in Chart 1, judges made 44 suggestions, and only three were not met after the research team met, such as using the term “resuscitation” instead of “reanimation”; using scissors to cut clothes to expose the victim’s chest; and using Advanced Cardiac Life Support (ACLS) guidelines to guide the steps of cardiopulmonary resuscitation. The justifications for the three refusals were presented in the discussion topic.

The ten lay judges who participated in the storyboard’s phase and content validity were predominantly male (n=7), under 30 years old (n=9) and with high school education (n=6). In the group of 10 expert judges, there was a predominance of women (n=7), aged 30 years and older (n=8), with a nursing degree completed between 2010 and 2020 (n=8), all judges were nurses. Most worked in the care area for more than 5 years (n=8) and specialized in emergency (n=4), intensive care (n=4) and cardiology (n=2).

To confirm the agreement among judges, the Content Validity Index was performed for each assessed scene (Table 1). The result obtained met expectations in validating the storyboard face and content, since its mean in each group was ≥ 0.90.

**Table 1:** Content Validity Index values according to judges’ assessment for each storyboard scene, Chapecó, Santa Catarina, Brazil, 2022

Questions	Content Validity Index		
	Lay judges	Expert judges	All judges
C1	0.90	0.90	0.90
C2	1.00	0.70	0.85
C3	0.90	0.90	0.90
C4	1.00	1.00	1.00
C5	1.00	1.00	1.00
C6	1.00	0.90	0.95
C7	1.00	0.90	0.95
C8	1.00	1.00	1.00
C9	1.00	0.80	0.90
C10	0.90	0.90	0.90
C11	0.90	0.90	0.90
C12	1.00	1.00	1.00
C13	1.00	0.80	0.90
C14	1.00	0.90	0.95
**Mean CVI**	0.97	0.90	0.94

For socialization purposes, use the cell phone camera to read the QR CODE below and watch the video produced, free of charge.

**Figure 1:**
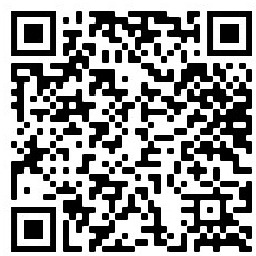
QR-CODE for access to the “Educational Video for Training in Cardiopulmonary Resuscitation with Chest Compressions Only in Adults”, Chapecó, Santa Catarina, Brazil, 2022

## DISCUSSION

Storyboard face and content validity reached the desired parameters, since its Content Validity Index met the recommended by the literature. Nursing researchers^(12)^ describe that using the Content Validity Index is one of the most used in studies in the health area. Recently, using this index has been criticized by some authors who believe that there is an overestimation of agreement between judges. This situation would be the result of the interference of random factors that would limit evaluators’ criticality. They point out that the presence of a high criticality index would be important when judges were analyzing the validity of construct measurement instruments^(13)^. We understand that the Content Validity Index was adequate for the present study, whose objective was to validate a storyboard and, subsequently, create an educational video, not a construct measurement instrument. Based on previous experiences in educational video construction, the assessment of judges’ agreement was not restricted to the proposed index, and the evaluators were offered a space to insert their comments, which were also part of the final analysis of the storyboard.

In addition to statistics, the logical sequence of the scenes and the layout of their instructional content were produced with accessible and objective language for their target audience: lay people. In the scientific literature, it is possible to identify video production for teaching cardiopulmonary resuscitation for laypeople, as carried out by Spanish researchers^(14)^, but production has sports as its setting as well as it is not possible to identify information about its validity. In a similar study^(15)^, despite achieving positive results in training lay people, the video was validated only by a professional category, without the contribution of its target audience. It is considered that their participation strengthens content structure, and consequently, contributes to learning.

Researchers^(16)^ produced an educational video about newborn care, and, in the production phase, associated the assessment of more than one group of judges, including the target audience. Such a strategy was interpreted as enhancing participants’ learning, in the next step, to assess the acquired knowledge. It is worth mentioning that the Content Validity Indexes among judges were ≥ 0.90, similar to the present investigation. It reinforces that videos are excellent strategies for training lay people^(17)^, but they need methodological rigor in their production^(18)^.

In the present study, we have evidence that the storyboard created had face and content validity when assessed by health professionals and lay people. Despite the fact that the selection criteria for participants contain limitations on the training of expert judges in nursing and medicine, we did not have the participation of medical professionals. It should be noted, however, that nurses who acted as judges had qualifications and professional experience in the area addressed, fundamental characteristics for the assessment of the storyboard. In the production of the video, we chose to focus on training for chest compressions during cardiopulmonary resuscitation. This decision was based on the AHA guidelines^(3)^, which indicate that lay rescuers should only perform chest compressions in situations of cardiorespiratory arrest. In this regard, it is important to highlight that during the storyboard composition, the presentation of cardiopulmonary resuscitation maneuvers was divided into steps that were didactically understandable to viewers. It is noteworthy that, in the real experience of a cardiorespiratory arrest, the time between one step and another is short and they can occur simultaneously, as in the case of scenes 6, 7 and 8, which address the moment of contact with SAMU 192 and initiation of cardiopulmonary resuscitation. In a real situation, while a person calls for help to the health service, a trained lay person should already start cardiopulmonary resuscitation, as indicated by the AHA^(3)^.

An important aspect to highlight is that, in the video produced, all the actors involved in cardiorespiratory arrest scenes are young, which apparently differs from the data presented by DataSUS, which indicate that cardiovascular conditions have a higher incidence in the age group of 60 to 69 years old^(19)^. However, it is believed that this age difference does not harm teaching the technique while watching the video, reaching lay people of different ages for health education.

In a study aiming to assess the effectiveness of educational videos in training lay people on cardiopulmonary resuscitation, the researchers found an improvement in the recognition of cardiorespiratory arrest, memorizing the telephone number of the emergency service, correct victim positioning and the strength required for the maneuver^(14)^. These aspects were emphasized in the video produced for this research.

It should be reiterated that the act of educating is conceptualized as an exchange of empirically established knowledge, as well as that scientifically proven, with effectiveness and clarity, i.e., that the exposed information is fully captured^(20)^. Health training for lay people permeates health education, with regard to the exposure of contents that enable self-care or care for others in an emergency way and without causing damage to health, a factor that impacts on patients’ survival^(21)^. However, video production with the aim of educating in health, when elaborated without scientific knowledge, even by health professionals, or produced by lay individuals, generate the opposite result to the aforementioned information, and provide inefficient and/or harmful actions for those who consume them. Therefore, health content with an instructive or training content on media platforms must include scientifically proven evidence, as they minimize the risks of miseducation^(22-23)^.

With regard to the consolidation of information presented in the storyboard, three of judges’ suggestions were not met by the research team, namely: using the term “resuscitation” instead of “reanimation”; using scissors to cut clothes to expose the victim’s chest; and using ACLS guidelines to guide the cardiopulmonary resuscitation stages. It is justified that discussions about using the term “resuscitation” instead of “reanimation” are old in Brazil. It is believed that “reanimation” is the most appropriate term, since “resuscitation” alludes to the magical and religious context of bringing back to life^(24)^. In the Health Sciences Descriptors (DeCS) of the Latin American and Caribbean Literature in Health Sciences (LILACS) database, the standardized term is “cardiopulmonary resuscitation”, however “cardiopulmonary resuscitation” appears as a synonym, which means that the term is still commonly used, in addition to being recognized by the scientific community. Therefore, opting for one term or another is up to the interlocutor, as in the case of the authors of this research. The inclusion of use of scissors to cut clothes becomes impracticable in everyday life and optional in the training of lay people^(1)^. Regarding ACLS guidelines, it should be noted that such training aims to regulate and standardize the necessary actions to be carried out by health professionals in the intra-hospital environment, with no relevance in the training of lay people.

Finally, the current ease of disseminating videos over the internet expands the reach of lay people who are willing to learn about cardiopulmonary resuscitation. Researchers^(25)^ identified that educational videos made available on virtual platforms reached more than thirty thousand people, including Brazilians and foreigners, demonstrating, once again, its potential in health education for laypeople. In another investigation, the authors^(26)^ show that after the SARS-CoV-2 virus pandemic, there was a relevant increase in the search for online health training on the various video and videoconference platforms. Therefore, it is believed that the use of audiovisual resources is growing in the various dimensions of health education.

### Study limitations

The study carried out does not include other necessary steps to analyze the educational video face and content validity, such as functionality and usability^(27)^, which respectively assess whether the technology produced meets the needs of its users and whether they are able to identify the information provided and use it correctly. Such steps will be conducted later by the researchers involved in the present study.

### Contributions to nursing, health or public policies

The video produced in this investigation will serve as one of the subsidies used by nurses, and other health professionals, for the training of lay people who transit through health care spaces or not. In composing the storyboard, we sought to describe the step-by-step steps of cardiopulmonary resuscitation, with emphasis on chest compressions, with a view to reducing this limitation and providing fundamental information so that spectators know how to act in this adversity. It is also worth highlighting that the video produced does not extinguish the need for face-to-face training in Basic Life Support.

The scientific production of easily accessible and quickly disseminated audiovisual material, in times of fake news, represents the academy’s social responsibility by promoting health with science.

## CONCLUSIONS

Educational health video production is a challenge nowadays, mainly due to the cost involved in production and its scarcity in the academic environment. However, they are necessary to educate in the digital age, either in the professional context or for training lay people, as proposed in this research. The educational video produced, focusing on training laypersons for cardiopulmonary resuscitation using only chest compressions, met the proposed objectives, since the methodological path, was developed in accordance with the adopted frameworks, in addition to its storyboard having been validated by judges as to its face and content. Therefore, the material has both scientific information and expertise of health professionals and the perceptions of the target audience itself, lay people, so it can be said that the product of this investigation is important scientific evidence and material to be used in health education practice.

## References

[1] Bernoche C, Timerman S, Polastri TF, Giannetti NS, Siqueira AWS, Soeiro APAM (2019). Atualização da Diretriz de Ressuscitação Cardiopulmonar e Cuidados Cardiovasculares de Emergência da Sociedade Brasileira de Cardiologia. Arq Bras Cardiol.

[2] Zandomenighi RC, Martins EAP. (2018). Análise epidemiológica dos atendimentos de parada cardiorrespiratória. Rev Enferm UFPE.

[3] American Heart Association (2020). Estados Unidos da.

[4] Arnemann CT, Lavich CRP, Terra MG, Mello AL, Raddatz M. (2018). Educação em saúde e educação permanente: ações que integram o processo educativo da enfermagem. Rev Baiana Enferm.

[5] Fernandes JR. (2019). Educação em saúde: o papel do enfermeiro como educador em saúde no cenário de IETC. Rev JOPIC [Internet].

[6] Porto JS, Marziale MHP. (2020). Construction and validation of an educational video for improving adherence of nursing professionals to standard precautions. Texto Contexto Enferm.

[7] Fleming SE, Reynolds J, Wallace B. (2009). Lights... camera... action! a guide for creating a DVD/video. Nurse Educ.

[8] Panchal AR, Bartos JA, Cabañas JG, Donnino MW, Drennan IR, Hirsch KG (2020). Part 3: Adult Basic and Advanced Life Support: 2020 American Heart Association Guidelines for Cardiopulmonary Resuscitation and Emergency Cardiovascular Care. Circulation.

[9] Pasquali L. (1997). Psicometria: teoria e aplicações.

[10] Cruz FOAM, Faria ET, Reis PED. (2020). Validation of an educational manual for breast cancer patients undergoing radiotherapy. Rev Latino-Am Enfermagem.

[11] Cavalcante LDW, Oliveira GOB, Almeida PC, Rebouças CBA, Pagliuca LMF. (2015). Assistive technology for visually impaired women for use of the female condom: a validation study. Rev Esc Enferm USP.

[12] Alexandre NMC, Colucci MZO. (2011). Validação de conteúdo nos processos de construção e adaptação de instrumentos de medidas. Ciênc Saúde Coletiva.

[13] Almanaresh E, Moles R, Chen TF. (2019). Evaluation of methods used for estimating content validity. Res Social Adm Pharm.

[14] Alvarez-Cebreiro N, Abelairas-Gómez C, García-Crespo O, Varela-Casal C, Rodriguez-Nuñez A. (2020). Efecto de la formación en soporte vital básico a través de un video difundido en redes sociales. Educ Méd.

[15] Araújo DV, Sampaio JVF, Oliveira IKM, Silva JA, Galindo NM, Barros LM. (2021). Efetividade de vídeo educativo no conhecimento de leigos em sala de espera sobre a reanimação cardiopulmonar. Enferm Actual Costa Rica.

[16] Sousa LB, Braga HFGM, Alencastro ASA, Silva MJN, Oliveira BSB, Santos LVF (2022). Effect of educational video on newborn care for the knowledge of pregnant and postpartum women and their families. Rev Bras Enferm.

[17] Siqueira TV, Nascimento JSG, Regino DSG, Oliveira JLG, Pereira LA, Dalri MCB. (2021). Estratégias educativas de ressuscitação cardiopulmonar para leigos: revisão integrativa da literatura. Rev Mineira Enferm.

[18] Elangovan S, Kwan YH, FonG W. (2020). The usefulness and validity of English-language videos on YouTube as an educational resource for spondyloarthritis. Clin Rheumatol.

[19] Ministério da Saúde (BR) (2022). DataSUS. TabNet: informações de saúde[Internet].

[20] Moraes MC, Betalloso JM. (2018). Transdisciplinaridade, criatividade e educação: fundamentos ontológicos e epistemológicos.

[21] Landa J, Ferreira AMGB. (2020). Transferência do Conhecimento de Suporte Básico de Vida para Leigos e Profissionais de Saúde: uma revisão integrativa. Rev Bras Multidisc.

[22] Dalpoz GQ, Higasi MS, Uchida TH, Fujimaki M. (2022). Analysis of YouTube® educational videos on prevention of dental caries. Res Soc Develop.

[23] Silva BWAC, Araújo AKD, Ó LB, Medeiros MBC, Melo VL, Sena JF (2020). Analysis of self-care videos on YouTube about exchange of intestinal ostomy bags. Rev Rene.

[24] Guimarães HP, Lane JC, Flato UAP, Timerman A, Lopes RD. (2009). Uma breve história da ressuscitação cardiopulmonar. Rev Bras Clín Méd [Internet].

[25] Libardi MBO, Duarte JMO, Lima JAF, Monteiro SNC, Vaz TS, Torri Z. (2018). Comunicação em saúde por meio do ambiente virtual: relato de experiência. Rev Gaúcha Enferm.

[26] Amaral MM, Rossini TSS, Santos EO. (2021). Viralização da educação online: a aprendizagem para além da pandemia do novo coronavírus. Práxis Educ.

[27] Cruz FOAM, Faria ET, Ghobad PC, Alves LYM, Reis PED. (2021). A Mobile App (AMOR Mama) for Women With Breast Cancer Undergoing Radiation Therapy: functionality and usability study. J Med Internet Res.

